# Fundamental processes in sensorimotor learning: Reasoning, refinement, and retrieval

**DOI:** 10.7554/eLife.91839

**Published:** 2024-08-01

**Authors:** Jonathan S Tsay, Hyosub E Kim, Samuel D McDougle, Jordan A Taylor, Adrian Haith, Guy Avraham, John W Krakauer, Anne GE Collins, Richard B Ivry

**Affiliations:** 1 Department of Psychology, Carnegie Mellon University Pittsburgh United States; 2 Neuroscience Institute, Carnegie Mellon University Pittsburg United States; 3 School of Kinesiology, University of British Columbia Vancouver Canada; 4 Department of Psychology, Yale University New Haven United States; 5 Department of Psychology, Princeton University Princeton United States; 6 Department of Neurology, Johns Hopkins University Baltimore United States; 7 Department of Psychology, University of California Berkeley Berkeley United States; 8 Helen Wills Neuroscience Institute, University of California Berkeley Berkeley United States; 9 Department of Neuroscience, Johns Hopkins University Baltimore United States; 10 Santa Fe Institute Santa Fe United States; Western University Canada; University of California, Los Angeles United States

**Keywords:** motor learning, expertise, cognitive science, motor control, learning, skill acquisition

## Abstract

Motor learning is often viewed as a unitary process that operates outside of conscious awareness. This perspective has led to the development of sophisticated models designed to elucidate the mechanisms of implicit sensorimotor learning. In this review, we argue for a broader perspective, emphasizing the contribution of explicit strategies to sensorimotor learning tasks. Furthermore, we propose a theoretical framework for motor learning that consists of three fundamental processes: reasoning, the process of understanding action–outcome relationships; refinement, the process of optimizing sensorimotor and cognitive parameters to achieve motor goals; and retrieval, the process of inferring the context and recalling a control policy. We anticipate that this ‘3R’ framework for understanding how complex movements are learned will open exciting avenues for future research at the intersection between cognition and action.

## Motor skill acquisition involves both implicit learning and explicit strategy

**Figure 1.**
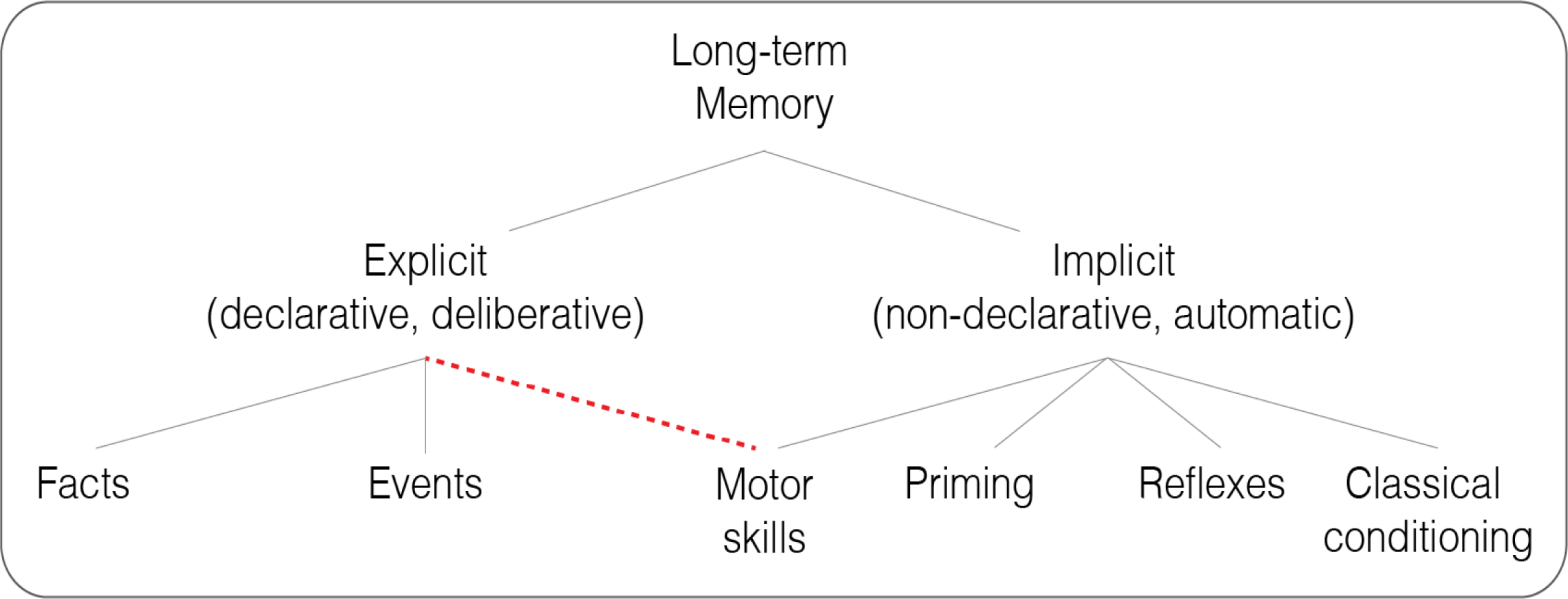
Classic and revised taxonomies of long-term memory. A revision of the classic taxonomy proposed by Squire and Zola, 1996 (gray lines), with motor skills tapping into both explicit and implicit memory (dashed red line).

Leaf through any neuroscience textbook and motor learning – the process of refining our movements through feedback and practice – will be described as an implicit, non-declarative phenomenon (Figure 1). Indeed, this description matches the phenomenology of skilled performers who ‘let the body do the thinking’ when executing a highly practiced motor skill (Jackson, 1996). In the domain of cognitive psychology, this perspective can be traced back to the foundational studies with patient H.M., an individual who had undergone bilateral medial temporal lobectomy and subsequently developed severe anterograde amnesia (Scoville and Milner, 1957). Despite having no conscious recollection of performing a mirror drawing task, H.M. exhibited striking improvements over multiple sessions of practice (Milner, 1962). This monumental finding helped inspire taxonomies of human learning and memory that place motor skill learning (sometimes called ‘procedural learning’) squarely in the domain of implicit memory (Squire, 2004; Squire and Zola-Morgan, 1991).

This simplified and intuitive perspective overlooks a crucial distinction: while H.M. may lack *explicit memory* of what he had learned between sessions, he may well have employed *explicit strategies for learning* within each session (Krakauer et al., 2019; McDougle et al., 2022b). Recent research provides compelling evidence in support of this hypothesis, showing not only the operation of multiple learning processes during mirror drawing, but also that the explicit component of learning is the primary impetus for improvement (Wilterson and Taylor, 2021). More generally, it would be difficult to find a motor skill that does not require the application of explicit strategies (Stanley and Krakauer, 2013).

## The contribution of strategy use in simple sensorimotor learning tasks

How do we draw the boundaries between implicit learning and explicit strategy? Implicit learning plays a crucial role in ensuring well-calibrated movements, a process that operates automatically and outside of conscious awareness (Mazzoni and Krakauer, 2006; Morehead et al., 2017; Tsay et al., 2020). Conversely, explicit strategies are movement plans that are *reportable* (explicit) and/or *intentional* (strategic) (Deng et al., 2022; Hegele and Heuer, 2010; Kim et al., 2021; Lillicrap et al., 2013; Maresch et al., 2021; McDougle et al., 2016b; Morehead and Orban de Xivry, 2021; Seidler and Carson, 2017; Sülzenbrück and Heuer, 2009; Taylor et al., 2014; Werner et al., 2015). Specifically, a reportable motor plan is one that participants can articulate, whereas an intentional motor plan is cognitively demanding and executed deliberately. For the purposes of this article, we will often use the terms ‘*explicit’* and ‘*strategy’* interchangeably (see Box 1).

Box 1. **Taking aim toward definitions of implicit and explicit motor learning.**

**Figure 2.**
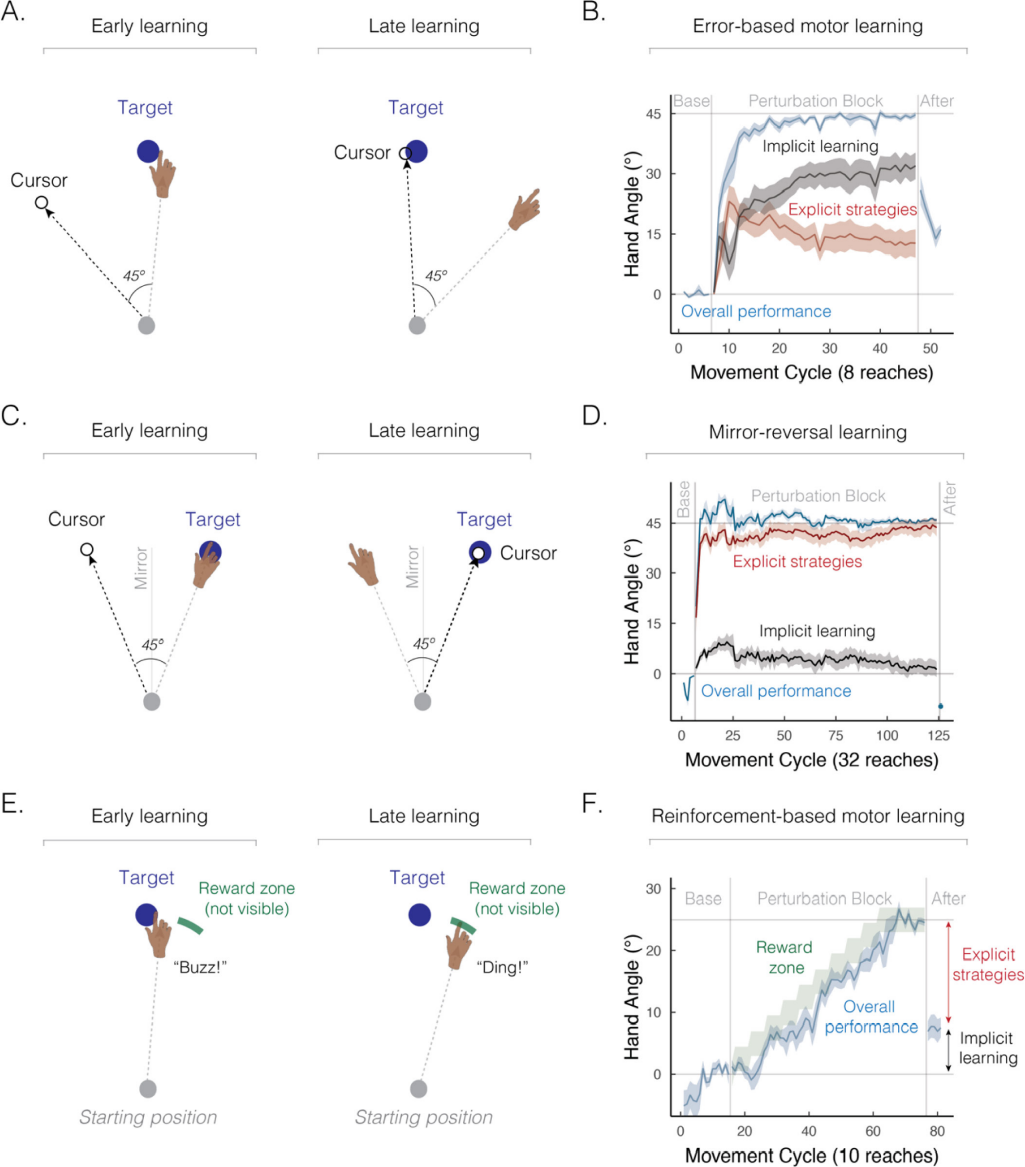
Implicit and explicit learning processes contribute to a wide range of sensorimotor learning tasks. (**A**) Schematic of an error-based motor learning task. The 45° rotated cursor feedback (white dot) was provided throughout the movement. (**B**) Mean time course of hand angle (light blue line) during baseline, perturbed feedback, and aftereffect (no feedback) phases. Red line denotes the time course of strategy use, measured by verbal reports of the aiming location using a number wheel. Black line denotes the time course of implicit learning, estimated by subtracting verbal reports of aiming location from overall performance. Hand angle is presented relative to the target (0°). Figure adapted from Figure 2C in Taylor et al., 2014. (**C**) Schematic of a mirror-reversal task. The visual cursor feedback (white dot) was reflected over the vertical axis and provided throughout the movement. (**D**) Mean time course of hand angle in a mirror reversal task. Figure adapted from Figure 10B in Wilterson and Taylor, 2021. (**E**) Schematic of a reinforcement-based motor learning task. A pleasant auditory ‘ding’ was provided when the movement passed within the reward zone (green arc); otherwise, an unpleasant ‘buzz’ was played. (**F**) Mean time course of hand angle during the reinforcement learning task. The reward zone (green zone) was gradually shifted, leading to learning (light blue line). Figure adapted from Figure 2A–B in van Mastrigt et al., 2023.

The two-pronged criteria of explicit strategy – reportability and/or intentionality – underpin many psychophysical methods used to measure this construct. In the aim report task (Taylor et al., 2014; Figure 2B), participants are asked to verbalize their intended motor plan for aligning a rotated cursor with a target. The difference between the location of verbal reports and the actual target location is a measure of reportable explicit strategy. In the ‘forced preparation task’ (Huberdeau et al., 2019; McDougle and Taylor, 2019), the time allowed for participants to prepare a movement is manipulated. The difference between trials with short or long preparation times provides a measure of intentional explicit strategy (Figure 2F; see Maresch et al., 2021 for an in-depth review).

We recognize that the two-pronged criteria of explicit strategy measurement may oversimplify the construct in several critical aspects. The terms 'implicit' and 'explicit' learning can have different definitions across different domains of learning. In statistical learning, 'implicit' usually denotes the process through which participants acquire through exposure/repetition the probabilistic structure of a task, whereas 'explicit' relates to the process where the structure of the task is transparently communicated to the participants (Reber, 1989). In sports psychology, these terms often refer to the necessity of attentional resources: implicit learning requires minimal attention and is resilient to dual-task interference, while explicit learning requires significant attentional resources, and is impacted by multi-tasking (Masters, 1992). Given the range of definitions, future research should work toward a consensus on a unified definition of these processes that account for the full range of replicable empirical features.

The reportability and intentionality of a movement may vary throughout the time course of motor skill acquisition. A learner might be able to describe how they are refining their movements (e.g., a biker can articulate *how* they lean into the turn to maintain stability at high speeds) but cannot report the underlying reason *why* their actions lead to a desirable outcome (e.g., the biker may fail to articulate the principles of centrifugal force). Moreover, when a strategy is completely stored in long-term memory, participants may find themselves unable to verbalize or exert any deliberate control over it, rendering it a form of implicit motor memory.

It is important to recognize that the extent to which strategies are reportable and/or intentional may exist on a continuum rather than a dichotomy. Participants might articulate a strategy with varying levels of confidence (Fassold et al., 2023; Hewitson et al., 2023; Yokoi and Weiler, 2022) or require degrees of deliberation depending on the cognitive resources available (Haith et al., 2015; Song and Bédard, 2015).

While explicit strategy use is commonly viewed as a singular concept, we foresee future research offering a clearer definition and an in-depth perspective on the various components, processes, and continua that underpin the construct.

Various forms of feedback and sensorimotor experiences, such as motor error, reward, and movement repetition, have traditionally been thought to induce implicit learning (Doya, 2000; Izawa and Shadmehr, 2011; Pascual-Leone et al., 1993; Shadmehr et al., 2010). Moreover, these forms of implicit learning are believed to depend on separable neural pathways: error-based motor learning engages cerebellar–cortical interactions (Marr, 1969), reinforcement-based learning engages basal ganglia–cortical interactions (Schultz et al., 1997), and use-dependent learning (i.e., learning driven by simple movement repetition) modulates neural tuning curves in primary sensorimotor areas (Classen et al., 1998; also see Areshenkoff et al., 2023; Nick et al., 2024; Standage et al., 2023). In this section, we discuss studies that have challenged the view that sensorimotor learning is solely implicit, demonstrating that performance on a broad range of simple motor learning tasks can be largely driven by the deployment of an explicit strategy.

### Error-based motor learning

The process of correcting motor errors via sensory feedback has proven to be a useful test bed for characterizing the contribution of both explicit and implicit learning processes (Anguera et al., 2010; Benson et al., 2011; Bromberg et al., 2019; Coltman et al., 2021; de Brouwer et al., 2018; Haith et al., 2015; Huberdeau et al., 2015; Kim et al., 2021; Taylor et al., 2014). Traditionally, error-based learning has been characterized by gradual implicit changes in movement kinematics, such as the heading angle of the hand in response to perturbed sensory feedback during goal-directed reaching tasks (e.g., Figure 2A; a rotation of visual feedback; Held and Hein, 1958; Helmholz, 1909). These implicit changes in reach kinematics remain robust even when the perturbed sensory feedback is removed, and, in fact, persist when participants are instructed to reach directly toward the visual target, forgoing the use of any strategy. In essence, this form of implicit learning induces a kind of visuomotor illusion, where one thinks that they are moving in one direction (e.g., reaching straight ahead) but are actually moving in an adapted manner (e.g., reaching 20º clockwise of their intended movement direction). This type of mismatch, dubbed an ‘aftereffect’, is a canonical signature of implicit learning.

However, two key pieces of evidence highlight the fact that explicit strategies contribute significantly to error-based motor learning. First, while participants can successfully adapt to large perturbations, such as a visual rotation of 45°, aftereffects are considerably smaller (e.g., around 15–25º on average), consistent with the hypothesis that only a part of the observed learning is truly implicit (Figure 2B). Second, when people are asked to verbally indicate where they intend to aim before each movement, the reports reveal that a large portion of learning is driven by explicit strategies (Bond and Taylor, 2015; Day et al., 2016; McDougle et al., 2017). These findings elevate error-based motor learning from a process placed squarely in the domain of implicit memory to one that also relies on explicit, declarative strategies.

Beyond visuomotor rotation learning, explicit strategies have also been shown to operate in other error-based adaptation tasks, such as saccade adaptation (Huang et al., 2017), force-field adaptation (Schween et al., 2020), target-jump adaptation (Leow et al., 2024; Sadaphal et al., 2022), prism adaptation (Leukel et al., 2015; Prablanc et al., 2020; Redding and Wallace, 2002), and locomotor adaptation (Ellmers et al., 2020; Malone and Bastian, 2010; Roemmich et al., 2016). The ubiquity of strategy use across such diverse paradigms speaks to its central importance in sensorimotor control.

### Mirror-reversal learning

The task used in the classic patient H.M. study is now recognized as one of the most compelling cases for strategy use (Ewert, 1930; Sekiyama et al., 2000; Stratton, 1897; Sugita, 1996; Telgen et al., 2014). Recent efforts have been made to quantify the relative contribution of implicit and explicit components to mirror-reversal learning (Figure 2C; Hadjiosif et al., 2020; Lillicrap et al., 2013; Wilterson and Taylor, 2021; Yang et al., 2021). Based on verbal reports about the intended aiming position, over 90% of learning can be attributed to explicit strategies (Figure 2D). Additionally, the substantial time required for movement planning (Wilterson and Taylor, 2021), as well as learning impairments observed under dual-task conditions (Eversheim and Bock, 2001), all indicate that mirror reversal learning relies heavily on strategy use.

Multiple learning processes also contribute to *reinforcement-based motor learning*, the process of refining movements through reward and/or punishment (Galea et al., 2015; Izawa and Shadmehr, 2011). In the initial work with this method, learning was assumed to occur primarily via implicit processes (Cashaback et al., 2019; Galea et al., 2015; Izawa and Shadmehr, 2011; Nikooyan and Ahmed, 2015; Uehara et al., 2019; Wu et al., 2014). However, reinforcement-based motor learning engages both implicit and explicit processes (Butcher and Taylor, 2018; Forano and Franklin, 2024; Holland et al., 2018; Kunavar et al., 2023): as illustrated in Figure 2E and F, participants can successfully adjust their movements based on binary reinforcement feedback signaling whether their movements hit or missed a hidden, and gradually shifting, reward zone (van Mastrigt et al., 2023). Strikingly, only a small portion of the learning arises from implicit processes as indexed by the aftereffect phase when participants are instructed to forgo strategy use and reach directly to the visual target.

Two additional pieces of evidence emphasize the presence of reinforcement-driven sensorimotor strategies. First, unlike error-based sensory feedback (Block and Bastian, 2011; Ruttle et al., 2021; Tsay et al., 2021b), binary reinforcement does not distort the participants’ sense of hand position (Izawa and Shadmehr, 2011), strengthening the claim that the sensorimotor map is not implicitly recalibrated by reward and/or punishment. Second, learning is severely compromised when the task is performed concurrently with a secondary task, indicating that reinforcement-based motor learning is cognitively demanding (Codol et al., 2018; Holland et al., 2018). Together, these findings make a strong case for strategy use during reinforcement-based motor learning.

**Figure 3.**
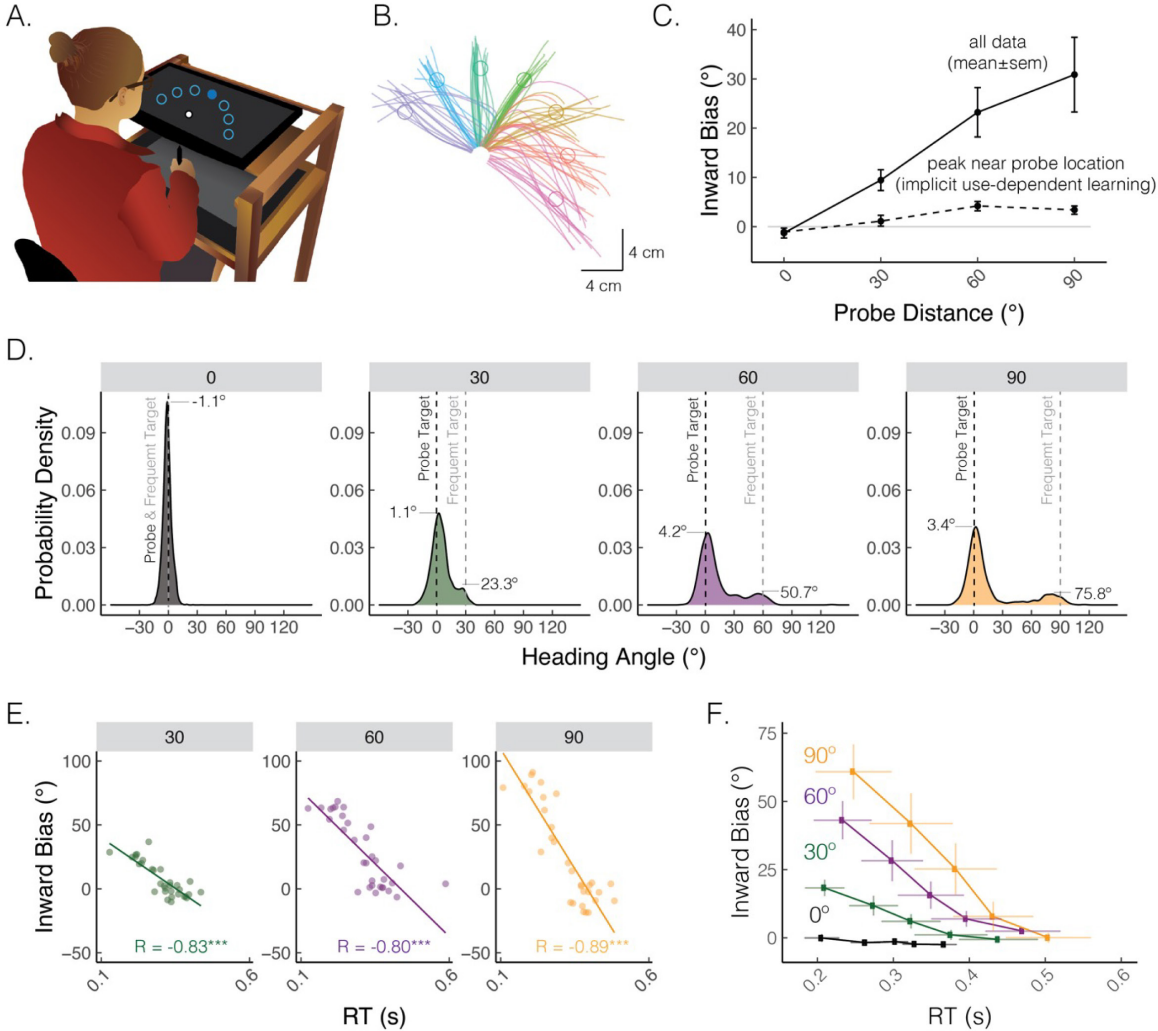
A default motor plan toward a repeated movement direction can be strategically overridden. (**A**) Reaching set-up showing locations of frequent and rare probe targets. Only one of seven targets (filled blue circle) was visible on each trial. (**B**) Movement trajectories from a representative participant who exhibited straight and curved movements. The frequent target is denoted by the green circle, whereas rare targets are denoted by the purple, blue, turquoise, yellow, red, and magenta circles (clockwise). (**C**) Average inward bias measured early in movement (at peak velocity) increased as a function of probe distance (solid line). By contrast, the peak of the Gaussian estimated from the distribution near the probe location saturates for larger probe distances (dashed line). (**D**) Distribution of heading angles for each of the probe distances. Gray dashed line denotes the location of the frequently presented target, and black dashed line (0 on the x-axis) denotes the location of the probe target. The means obtained from the mixture of Gaussians models are provided. (**E**) Bias as a function of reaction time (RT) for a representative participant. Dots indicate individual reaches, with the thin line showing the best-fitting regression line. R denotes Pearson correlation; ^***^p<0.001. (**F**) Group-level analysis of bias as a function of RT. For each individual, RTs were binned into quintiles and mean bias was calculated for each quintile. These data were then averaged across the group. Error bars denote SEM. Figure adapted from Figure 1 in Tsay, Kim et al. (2022).

Multiple learning processes also play a role in *use-dependent motor learning*, the process of refining movements through repeated practice, independent of feedback (Figure 3A and B; Classen et al., 1998; Mawase et al., 2017). In reaching studies, use-dependent learning is evident as a bias toward a frequently performed movement direction (Figure 3C; Diedrichsen et al., 2010; Verstynen and Sabes, 2011). This movement bias has been assumed to be implicit and rigid, meaning it could not be deliberately overridden by explicit processes. However, recent findings have demonstrated that a large portion of use-dependent biases can be strategically reduced (Marinovic et al., 2017; Reuter et al., 2019; Suleiman et al., 2023; Tsay et al., 2022; Wong and Haith, 2017): as illustrated in Figure 3D–F, a use-dependent bias toward a frequently repeated movement direction is much more pronounced for faster and more impulsive movements, reflecting a ‘default’ motor plan that is chosen based on experience repeating the same movement. Strikingly, the bias is nearly abolished when movement initiation is slowed down. These findings reveal that moving to a relatively rare target location may require strategic re-aiming to override a default motor plan directed toward the frequent target location. Indeed, the implicit component of use-dependent biases, which manifests when movement initiation is slowed down, is quite small (<5º).

Beyond the sensorimotor learning tasks outlined above, consideration of multiple processes is also important for understanding *motor sequence learning* (Krakauer et al., 2019). The serial reaction time (SRT) task has been widely deployed as a test of implicit learning. However, even the earliest studies using the SRT task demonstrated that explicit learning can play a major role in performance, impacting how participants represent the structure of the sequence (Cohen et al., 1990; Jiménez et al., 2006; Nissen and Bullemer, 1987). Moreover, even under conditions designed to minimize explicit learning, participants, including those with severe anterograde amnesia, can explicitly report sequence fragments. This explicit knowledge is, in fact, essential for significant performance improvements, accounting for much of the reduction in reaction time (Moisello et al., 2009; Reber and Squire, 1998; Reber and Squire, 1994).

## Implicit and explicit learning exhibit contrasting properties in simple visuomotor tasks

**Figure 4.**
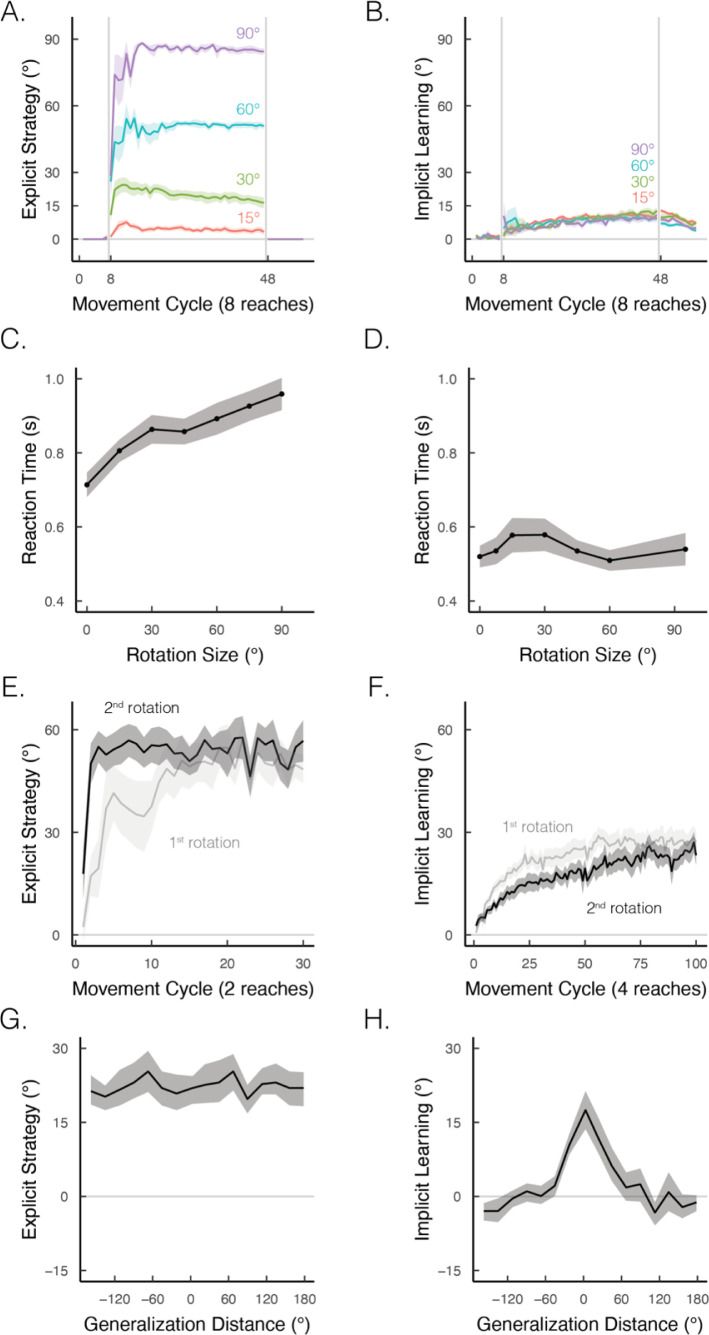
Implicit and explicit learning exhibit contrasting properties in visuomotor rotation tasks. (**A, B**) Learning functions requiring strategic re-aiming scale with the size of the rotation, whereas the size of the aftereffect, indicative of the implicit component, does not scale. Figure adapted from Figure 7 in Bond and Taylor, 2015. (**C, D**) When learning is under explicit control, reaction times scale with the size of the rotation; this effect is not observed when learning is implicit. Figure adapted from Figure 2B in McDougle and Taylor, 2019 and Figure 4 in Morehead et al., 2017. (**E, F**) Re-exposure to the same rotation enhances explicit learning but attenuates implicit learning. Figure adapted from Figure 1A in Tsay, Schuck et al. (2022) and Figure 2D in Avraham et al., 2021. (**G, H**) Explicit learning generalizes globally over the entire workspace, whereas generalization from implicit learning is local, centered around the aiming location (i.e., 0° on the x-axis). Figure adapted from Figure 2B in McDougle et al., 2017.

What are the key properties that distinguish explicit from implicit motor learning? In visuomotor rotation tasks, the two processes differ in terms of their responsiveness to task demands. Explicit strategy use is remarkably flexible, eliciting performance improvements that scale with the size of the rotation (Figure 4A; Bond and Taylor, 2015). In contrast, implicit learning is strikingly rigid, exhibiting similar performance across a wide range of rotation sizes (Figure 4B; Kim et al., 2018; Marko et al., 2012; Morehead et al., 2017; Tsay et al., 2021a; Wei and Körding, 2009). However, the flexibility associated with strategy use makes this process highly erratic. Not only can there be abrupt, dramatic changes in performance, but individuals sometimes employ strategies unnecessarily, even in the absence of a perturbation – a tendency that leads to self-induced motor errors (Miyamoto et al., 2020). Implicit learning can, to some extent, ‘clean up’ these self-induced errors, reinstating a more stable sensorimotor response.

By definition, the two processes differ in their cognitive demands: strategy use is intentional and deliberative, exhibiting reaction times that scale proportionally to the size of the rotation (Figure 4C; Fernandez-Ruiz et al., 2011; Huberdeau et al., 2015; McDougle and Taylor, 2019). Conversely, implicit learning is not cognitively demanding, showing similarly fast reaction times across a wide range of rotation sizes (Figure 4D; Hadjiosif et al., 2023; Haith et al., 2015; Leow et al., 2017; Morehead et al., 2017).

The two processes also differ in terms of savings and generalization. Strategy use is enhanced upon re-learning (i.e., savings; Figure 4E), whereas implicit learning is attenuated upon re-learning (Figure 4F; Avraham et al., 2021; Hadjiosif et al., 2023; Haith et al., 2015; Morehead et al., 2015; Tsay et al., 2024). Strategy use results in broad generalization to different target locations (Figure 4G; McDougle et al., 2017; McDougle and Taylor, 2019; Poh et al., 2021), exhibits almost full generalization when the movement is performed with a different effector than that used in training (Bouchard and Cressman, 2021; Werner et al., 2019), and is based primarily in extrinsic coordinate frames (Poh and Taylor, 2019). In contrast, implicit learning exhibits narrow generalization around the aiming location (Figure 4H; Day et al., 2016; Krakauer et al., 2000; Morehead et al., 2017), minimal generalization across effectors (Poh et al., 2016), and is based in both extrinsic and intrinsic coordinate frames (Brayanov et al., 2012; Poh and Taylor, 2019).

The timing of feedback has asymmetric effects on these two processes: while strategy use remains robust even when the feedback is significantly delayed (Brudner et al., 2016; Tsay et al., 2023b), implicit learning is very sensitive to the timing of feedback, relying on a close temporal association between movement initiation and feedback presentation (Kitazawa et al., 1995; Schween and Hegele, 2017; Wang et al., 2022). The effect of aging also has opposite effects on these two processes: while strategy use is markedly impaired in older adults, implicit learning is similar or even enhanced in older participants (Ruitenberg et al., 2023; Tsay et al., 2023a; Vandevoorde and Orban de Xivry, 2019; Vandevoorde and Orban de Xivry, 2020; Wolpe et al., 2020). This is consistent with the assumption that explicit strategy use relies on neural systems associated with planning and decision-making such as the prefrontal cortex and medial temporal lobe (Areshenkoff et al., 2023), regions that are most vulnerable to age-related decline (Persson et al., 2006; Wolpe et al., 2020).

**Figure 5.**
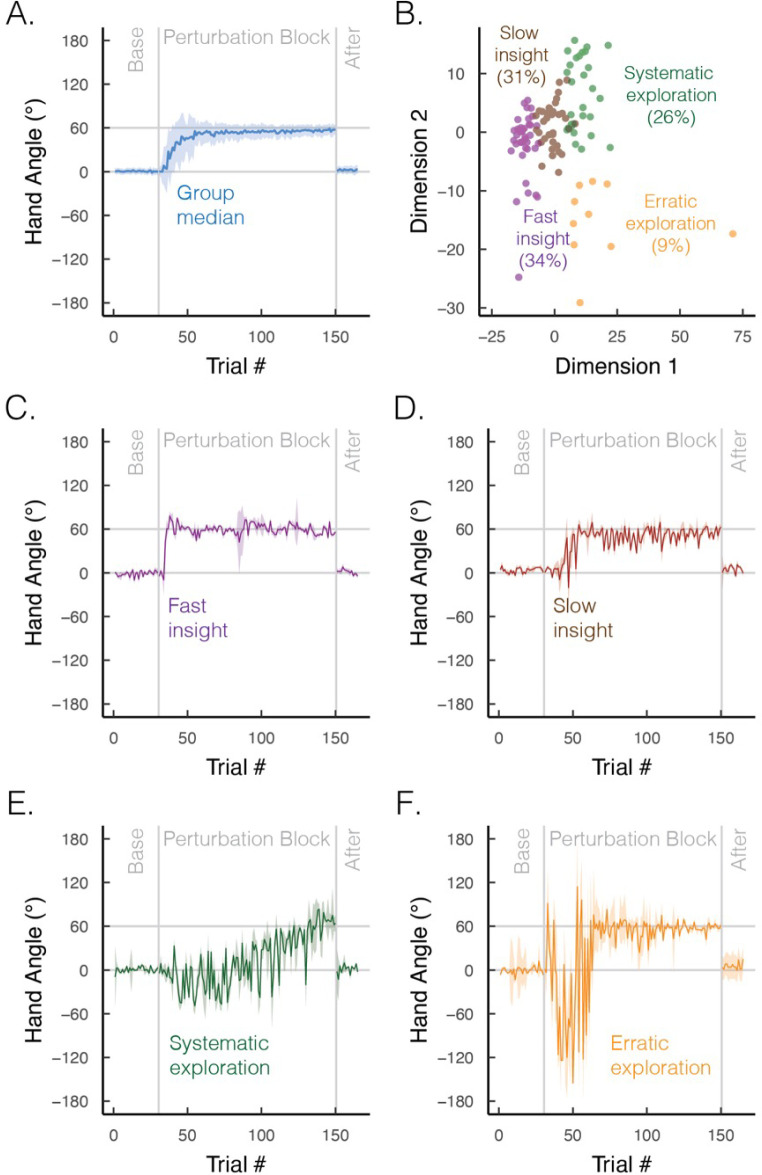
Group and subgroup performance in an explicit motor learning task. (**A**) Median time course of heading angle during baseline, perturbed feedback, and aftereffect (no feedback) phases. During the perturbation phase, the feedback cursor was rotated 60° from the actual position of the hand when the movement distance equaled the target amplitude. To isolate strategy use, we abolished implicit adaptation by delaying the onset of the feedback by 800 ms. The effectiveness of this manipulation is given by the fact that there is no evidence of an aftereffect. (**B**) Results from an unsupervised clustering algorithm. We used a dynamic time warping approach to quantify the degree of dissimilarity between participants’ time series (Jeong et al., 2011; Sidarta et al., 2022). We then applied a k-means clustering algorithm, which identified four optimal clusters via the elbow method of within-cluster sum of squared errors (colors denote different clusters; points denote different individuals). (**C–F**) Median time course of heading angle for the four subgroups that exhibit (**C**) fast insight, (**D**) slow insight, (**E**) systematic exploration (sign flips of ±60°), and (**F**) erratic exploration. Shaded error bars denote first/third interquartile range. Figures generated by data from Cisneros et al., 2024.

The time course of learning also varies between implicit and explicit processes. Implicit learning, at least in the motor domain, is well characterized by a process of gradual error reduction, a concept that has been central in computational models (Donchin et al., 2003; Fine and Thoroughman, 2007; Oh and Schweighofer, 2019; Shadmehr et al., 2010; Smith et al., 2006). In contrast, despite the appearance of gradual error reduction in the average learning curve (Figure 5A; figure adapted from Cisneros et al., 2024), individual learning functions are far more idiosyncratic on explicit learning tasks: some individuals exhibit sudden, punctuated jumps in performance (‘moments of insight’; Figure 5C and D), whereas others display large variabilities (‘exploratory patterns’; Figure 5E and F; McDougle and Taylor, 2019; Townsend et al., 2023). As such, instead of dismissing individual differences as mere noise, acknowledging these strategic differences may deepen our understanding of the diverse cognitive influences on motor learning.

However, there are two key considerations that temper our claims about contrasting features of implicit/explicit motor learning: first, while it is tempting to treat implicit and explicit motor learning processes as independent (Day et al., 2016; McDougle et al., 2017; Taylor et al., 2014), recent evidence suggests they interact in non-trivial ways (Albert et al., 2022; ’t Hart et al., 2023; Miyamoto et al., 2020; Therrien and Wong, 2021). For example, in traditional visuomotor rotation tasks, implicit learning appears to suppress strategy use (Shmuelof et al., 2012a; Vaswani et al., 2015; Weightman et al., 2022). This suppression may explain why participants fail to invoke a strategy to nullify a residual error that may persist after implicit learning has saturated; yet, in tasks where implicit learning is abolished (for instance, by significantly delaying the feedback), participants often succeed in strategically compensating for a similarly sized perturbation (Brudner et al., 2016; Tsay et al., 2023b; Wong et al., 2019). In another line of work, implicit and explicit learning are hypothesized to compete over a shared error signal (Albert et al., 2022). This competition may explain why manipulations that modulate strategy use (e.g., verbal instructions [Neville and Cressman, 2018] or perturbation schedule [Abeele and Bock, 2001; Kagerer et al., 1997; Modchalingam et al., 2023]) result in opposing effects on implicit learning. Taken together, we anticipate that research into how the properties of implicit and explicit learning differ when each process is studied in isolation versus when jointly operating will be an exciting avenue for future research.

Second, the dissociation between implicit and explicit motor learning processes has primarily been established in simple sensorimotor learning tasks, those in which most of the performance changes occur within just a few minutes. It remains to be seen whether analogous principles apply to the acquisition of complex motor skills – those that require hours, days, and even weeks to learn (Du et al., 2022; Haith et al., 2022; Listman et al., 2021; Nah et al., 2020; Scholz et al., 2009). We believe that understanding complex motor skill acquisition will pose a considerable challenge, one likely necessitating new computational principles and insights – a topic we will examine in the following section.

## The 3R framework for motor learning: Reasoning, refinement, and retrieval

Learning complex motor skills is a uniquely challenging endeavor. This process requires the simultaneous coordination of numerous muscles and joints, each subject to different biomechanical constraints and metabolic demands. While some constraints on coordination are wired into our genetic code and emerge spontaneously given the right environment, the coordination demands for complex skills will require considerable cognitive control over these many degrees of freedom. Additionally, learning complex motor skills often involves developing new physical intuitions about how subtle differences in movement can lead to drastically different outcomes (e.g., an amputee learning to use a new myoelectric prosthesis). Yet, despite these formidable challenges, humans are capable of learning a near-limitless repertoire of movements, enabling babies to walk and talk, athletes to achieve incredible levels of skill, and patients to recover from neurological disorders.

How humans achieve these impressive feats of complex motor skill acquisition remains poorly understood. This gap in knowledge partly stems from a long-standing neglect of the role of cognition in motor learning because such processes are generally hard to formalize and often exhibit high variability. Conversely, cognitive science frequently overlooks the role of motor control in decision-making; for example, many decisions are constrained by the sensorimotor outcomes associated with making a choice (Chen et al., 2017; McDougle et al., 2016a; Rmus and Zou, 2022). To make progress toward a comprehensive theory of motor learning, one that can explain the intricate cognitive–motor interactions that facilitate successful motor skill acquisition, we propose a ‘3R’ framework that integrates three fundamental concepts shared between the motor learning and cognitive science communities: reasoning, refinement, and retrieval. This new framework establishes a way to understand how new sensorimotor control policies – mappings between a learner’s state and context to an action – are acquired through reasoning, optimized through refinement, and automatized through retrieval.

### Reasoning

This involves understanding (often arbitrary) action–outcome relationships and using this knowledge to construct an effective control policy (Collins and Koechlin, 2012; Donoso et al., 2014; Heald et al., 2021; Lillicrap et al., 2013; Schone et al., 2023; Todorov and Jordan, 2002; Yang et al., 2021). To illustrate this concept, consider learning to ride a bicycle: one of the initial steps for the novice is to understand the relationship between the forces generated by the arms on the handlebars and the consequent movements of the bicycle (Table 1). Once the novice identifies the correct action–outcome relationship, she can leverage this physical intuition to derive a crude control policy (Allen et al., 2020; Battaglia et al., 2013; Fischer and Mahon, 2021).

**Table 1.** Reasoning, refinement, and retrieval in different motor skill learning contexts.

	Learning to bike	Learning to play tennis	Learning to play piano	Learning to walk
Reasoning	Understanding the mapping between arm movements and direction of the bike	Understanding how different arm and wrist movements affect the trajectory of the ball	Understanding the relationship between musical notes and the required finger movements	Developing an intuition for how to distribute weight to achieve balance
Refinement	Fine-tuning the amplitude of movement for smooth and efficient cycling.	Fine-tuning the angle of the stroke and racquet grip to accurately hit the ball	Fine-tuning the force and timing to enhance emotional expression	Fine-tuning the muscular coordination to maintain balance while walking
Retrieval	Performing a flawless ‘Wheelie Drop’ when encountering stairs.	Executing a complex spin serve when finding the opponent in a favorable receiving position	Improvising new musical pieces by combining learned melodies	Rapid recovery from stumbles

Motor learning researchers can draw valuable insights from cognitive science, a field that has had considerable success in formalizing computational models of reasoning. One flavor of reasoning is ‘Inference over Hypotheses’ (Griffiths et al., 2010; Piantadosi et al., 2016; Rule et al., 2020; Xia and Collins, 2021), which entails two main components: a hypothesis space and means to evaluate the elements of that space. For motor skills, the former might consist of domain-specific action primitives such as ‘push the right arm forward’ or ‘pull the left arm backward’ and relational primitives such as ‘or’, ‘and’, ‘before’, and ‘after’. By combining these primitives, more complex hypotheses can be created; for example, ‘pushing the right arm forward and pulling the left arm backward will move the bike leftward’. The merits of the hypotheses can be evaluated via inference, where learners use sensory feedback to strengthen or weaken their beliefs about each hypothesis.

Reasoning as inference has several advantages over previous ‘lower level’ models of motor learning. First, it can account for behaviors inconsistent with gradual error reduction. For example, marked exploratory behavior early in learning and punctuated jumps in performance may signify the rapid modification and adoption of new action–outcome hypotheses. During visuomotor rotation learning, errors may show systematic sign flips when the novice mistakes the direction of the perturbation as a clockwise rotation instead of a counterclockwise rotation. Or errors may systematically increase when a novice pursues an incorrect hypothesis such as mistaking a rotation for a mirror reflection. Second, reasoning as inference goes beyond learning which affine transformation (e.g., rotation, translation, reflection, etc.) best explains an action–outcome relationship (e.g., Baddeley et al., 2003; Burge et al., 2008; Garvert et al., 2023; Wei and Körding, 2010). Hypotheses may be more abstract in nature and thus comprise a near-infinite combination of action-relational primitives.

Reasoning can vary in the level of reportability and intentionality. Most often, reasoning is highly reportable and intentional (i.e., explicit and strategic). A learner might employ inferential reasoning to understand which set of primitives best explains the action–outcome relationship (e.g., “How should I best coordinate my arms to make a leftward turn on my bicycle?”) and/or abductive reasoning to identify the most plausible cause (e.g., “Did moving my right arm forward and left arm backward cause the bike to turn left?”). Reasoning may also be ineffable and unintentional, relying on computationally simpler and hypothesis-free approaches (Collins, 2018; Collins et al., 2017; Collins and Frank, 2012; Smith et al., 2023). For example, a learner may unconsciously adopt a simple win-stay/lose-shift heuristic, where successful actions are repeated, and unsuccessful actions are avoided. (It is debatable whether the use of heuristics constitutes reasoning or refinement.) Future studies are needed to precisely quantify how reasoning contributes to motor skill acquisition and explore whether and how reasoning processes help break down a complex skill into more learnable subcomponents.

### Refinement

This entails adjusting movement parameters to better achieve a motor goal. Here the term ‘motor goal’ is used to highlight the distinction between abstract goals related to decision-making (“I need caffeine.”) and those related to movement (“I am going to grasp the handle of the coffee mug.”) (Molinaro and Collins, 2023a; Wong et al., 2015). This refinement process is crucial as it enables learners to fine-tune their control policy to achieve movement goals with increased accuracy, precision, and efficiency. Building on the previous example, once our novice cyclist understands how manipulation of the handlebars controls the bike’s heading angle, she needs to refine this skill, learning the optimal timing and amplitude of the motor commands needed for different types of turns. Similarly, in visuomotor adaptation tasks, this refinement process is evident when motor behavior, initially abrupt and erratic, transitions to a more gradual and precise tuning of the angular shift required to nullify residual errors (Taylor and Ivry, 2011; Taylor et al., 2014).

Refinement can be viewed as a process of utility maximization (Wolpert and Landy, 2012; Yoon et al., 2020), with the inputs to the utility function dependent on task requirements. Classic models of motor learning have focused on maximizing sensorimotor utilities such as accuracy (Körding and Wolpert, 2004), precision (Shmuelof et al., 2012b), and energy conservation (Abram et al., 2019; Finley et al., 2013). A comprehensive model of strategy refinement will need to consider how both sensorimotor *and* domain-general utilities are jointly refined. Here, too, motor learning researchers can draw valuable insights from cognitive science. In that literature, domain-general utilities that constrain learning include reward (Sutton, 1999), intrinsic motivation (Kulkarni et al., 2016; Molinaro and Collins, 2023b; Wulf and Lewthwaite, 2016), financial incentives (Lebreton et al., 2018), cognitive effort (Frömer et al., 2021; Hodges and Lohse, 2020; Koranda et al., 2022), sense of agency (Haggard, 2017; Parvin et al., 2018), level of embodiment (Kieliba et al., 2021; Schone et al., 2023), informativeness (Barack et al., 2023), allocation of attention (Wulf and Su, 2007), and social praise (Mueller and Dweck, 1998).

While the control policy associated with an explicit strategy could be re-parameterized, this may be achieved by another round of reasoning. Refinement, therefore, primarily refers to the iterative, implicit adjustment to a control policy. Indeed, the process of implicit sensorimotor recalibration described in the section ‘The contribution of strategy use in simple sonsorimotor learning tasks’ could be considered a form of refinement since it typically leads to improved performance, with accuracy being the primary utility that is maximized (but see Mazzoni and Krakauer, 2006; Morehead et al., 2017).

### Retrieval

This entails recalling a control policy that has proven efficient in achieving a motor goal. Once a cyclist has refined the control policy for maintaining a steady bike ride, this policy becomes stored as a long-term motor memory and, with appropriate contextual cues, can be readily retrieved (Heald et al., 2021; Xia and Collins, 2021). For example, after many hours of practice, our bike rider when encountering a set of stairs might execute a flawless ‘Wheelie Drop’, lifting the front wheel off the ground to bounce down on just the rear wheel. Similarly, in visuomotor adaptation tasks, the retrieval process becomes evident as motor behavior transitions from reportable and intentional to ineffable and automatic (Haith et al., 2015; Huberdeau et al., 2019; McDougle and Taylor, 2019). With repetition, the retrieval of a control policy will no longer entail preparation costs (Huberdeau et al., 2019).

Importantly, this implicit process, which emerges from the proceduralization of a control policy (Anderson, 1982), differs significantly from that associated with implicit sensorimotor recalibration. First, a retrieved policy does not involve changes to a sensorimotor map, the mapping between the perceived and intended actions; as such, it will not result in an aftereffect, the cardinal signature of implicit recalibration. Instead, a retrieved policy is a well-practiced mapping between context/states and intended actions. Second, a retrieved policy can be reported and strategically overridden upon retrieval, particularly when individuals are given sufficient time (Hardwick et al., 2019; Werner et al., 2015). In contrast, implicit recalibration cannot be verbalized or deliberately overridden (Morehead et al., 2017; Tsay et al., 2020).

Cross-pollination between cognitive science (Cohen and Eichenbaum, 1993; Eckstein and Collins, 2020; Sanders et al., 2020; Xia and Collins, 2021) and motor learning (Haruno et al., 2001; Heald et al., 2021; Oh and Schweighofer, 2019) has fostered the development of several computational models of motor memory retrieval. These models formalize how learners use contextual information (e.g., sensory cues and bodily states) to retrieve the appropriate control policy for accomplishing a goal (Heald et al., 2021). These models generally consist of three components: first, the learner possesses a memory of various contexts, each associated with a control policy. For example, when heading out on a smooth well-paved trail, a mountain biker might adopt a narrow, aerodynamic position to increase speed, whereas to start down a rocky descent, shift to the back edge of the seat to adopt a more stable position. Second, the learner continuously makes contextual inferences from a stream of sensory cues. For example, if our biker starts feeling friction against her wheels, she might infer, with some uncertainty, that she is encountering a sandy or marshy section of the trail. If none of the contextual memories match the current context, the learner needs to create a new memory associated with a new control policy, one that can undergo further reasoning and refinement. Third, the learner selects an action based on an integrated control policy, one that may be derived by blending control policies and/or value functions associated with distinct contexts.

How does the 3R framework help us understand motor expertise? The emphasis on implicit retrieval over strategic reasoning bears similarity to instance theories of expertise in which practice results in the emergence of a large set of stimulus–response associations from which to draw on Logan, 1988. However, while such models might capture the accuracy and efficiency that comes with extended practice, they fall short in accounting for the flexibility that is also a hallmark of expertise. Instead, the 3R framework postulates that experts possess a wealth of contextual memories associated with a given motor task, each with a well-reasoned and well-refined control policy acquired through extensive practice and experience. Experts do not need to engage in the computationally demanding process of creating new memories and forming new control policies; their extensive sensorimotor repertoire allows them to identify the current context and recall the appropriate action efficiently and confidently. Moreover, should a motor error arise, the expert is well-poised to recognize whether the error originates from contextual inference, action execution, or action selection, with the latter quickly correctable by switching to another well-rehearsed control policy.

To illustrate how the 3Rs framework applies to learning another complex motor skill, let us examine the process of becoming proficient in tennis (Table 1). The reasoning phase is marked by an understanding of how different arm and wrist movements affect the racket’s swing and the ball’s trajectory. A beginner tennis player can explore different action–outcome hypotheses to determine which movements effectively control the racquet and direct the ball. The transition from the reasoning to the refinement phases signifies a shift from understanding which movements are appropriate for a given context to optimizing how the selected movements are performed to achieve specific motor goals. For example, a player aiming for accuracy might focus on fine-tuning the angle of both the stroke and racquet grip to accurately hit the ball. Conversely, a player striving for power might concentrate on larger, more forceful strokes. Finally, during the retrieval phase, the tennis player might note the position of their opponent when deciding where to place the serve and with which type of spin. This phase represents the pinnacle of learning, where complex actions are performed automatically and are seamlessly integrated into the player’s motor repertoire.

The 3R framework can also capture features of motor development. While the transition from sitting up to crawling to walking is certainly wired into our genes, variation across individuals and cultures suggest that the emergence of even such a fundamental skill may be shaped by reasoning, refinement, and retrieval (Campos et al., 1992). Children are unlikely to engage in complex forms of reasoning, but they may acquire motor skills through less cognitively demanding, heuristic-based forms of reasoning (Gopnik, 2012). For example, through practice and instruction, children might develop a better intuition for how to effectively shift their weight, aiding them in maintaining a more stable and balanced gait. Indeed, most learning likely arises from the implicit refinement of control policies, where they iteratively fine-tune the coordination between their upper and lower body muscles to achieve a more efficient gait. In the retrieval phase, the act of walking transitions from an effortful act to an automatic skill, enabling the child to walk more naturally and swiftly recover from stumbles.

How can we examine the neural correlates of motor skill acquisition? Although numerous approaches exist (Avila et al., 2022; Calame et al., 2023; Darmohray et al., 2019; Georgopoulos et al., 1989; Vyas et al., 2020), systems neuroscientists usually do not fully know the causal relationship between neural activity and behavior. Conversely, in a brain–computer interface (BCI) paradigm, the precise causal relationship between neural activity and behavior is determined by the BCI mapping, set by the experimenter. Frequently the initial mapping between neural activity to cursor movement is intuitive (e.g., neural population trajectories signaling a leftward arm movement moves the cursor leftward). However, new and arbitrary mappings can be introduced and readily learned (Carmena et al., 2003). As such, the BCI paradigm allows one to closely examine how changes in neural activity during learning lead to improved behavior.

Moreover, the BCI paradigm may reveal the neural circuits involved in reasoning, refinement, and retrieval (Golub et al., 2018; Hennig et al., 2021a; Hennig et al., 2021b). For example, reasoning may manifest as erratic changes in the animal’s kinematics during early learning (e.g., a cost in reaction time and/or systematic errors in movement direction). Using these data, we can infer the hypotheses the animal is pursing as well as examine how these hypotheses correlate with distinct neural trajectories, reflecting the range of action–outcome primitives. Refinement may manifest as gradual, iterative improvements in movement speed and accuracy. Using these data, one can explore whether refinement simply facilitates faster and more efficient changes in neural activity along the same neural trajectory or if it involves the evolution of novel neural trajectories (Losey et al., 2022). Finally, once control policies become implicit and are automatically retrieved, one can examine whether 'higher level' cortices remain necessary for action execution, or, similar to many reflexes, whether action execution has been offloaded to 'lower level' subcortical areas (Bartels et al., 2021). By adopting shared definitions and theoretical frameworks between human and animal researchers, we can accelerate our progress in understanding how reasoning, refinement, and retrieval are implemented by the motor system.

While future experiments are needed to probe the dynamics of reasoning, refinement, and retrieval throughout motor learning, these ideas should open exciting avenues to advance theories of skill acquisition and inform the design of training programs to enhance expertise.

## Comparing and contrasting the 3R framework with Fitts–Posner’s stages in motor skill learning

The 3R framework shares similarities with the classic skill acquisition framework proposed by Fitts and Posner, 1979. The Fitts–Posner framework describes three stages of learning: the cognitive, associative, and automatic stages. In the cognitive stage, the novice grasps an understanding of the goals of the task and the general structure of the actions required to achieve that goal. In the associative stage, the novice experiments with different gestures, learning the different movement subcomponents that form the skilled action. Finally, the automatic stage captures how the skill becomes refined, with the expert moving in an effortless and near-reflexive manner.

While the 3R and Fitts–Posner frameworks both acknowledge that the acquisition of motor skills involves a transition from being cognitively demanding to being more automatic, there are notable differences. First, the Fitts–Posner framework emphasizes a singular progression through the cognitive, associative, and automatic stages of learning. In contrast, the 3R framework starts from the premise that motor skills involve the operation of multiple learning processes, all of which may work in parallel. For example, when our tennis player reasons through how her arm and wrist movements impact the ball’s trajectory, she may be simultaneously refining her level of exertion, retrieving motor memories from her past badminton classes, and even implicitly recalibrating to the weight of her new racquet. This feature is crucial as it highlights that motor learning may not be a linear progression but rather a dynamic interplay of many processes evolving in parallel.

Second, the Fitts–Posner framework describes motor skill acquisition at a purely phenomenological level. In contrast, the 3R framework outlines specific computational mechanisms. For example, as a starting point, we suggested that reasoning might rely on inference and/or heuristics, refinement driven by utility maximization, and retrieval dependent on contextual inference. This level of computational specificity will hopefully inspire more concrete experimental tests, as well as facilitate easy integration with other learning processes. While reasoning, refinement, and retrieval constitute one route toward successful motor learning, these processes can be readily combined with other computational mechanisms, such as those for implicit error-based, implicit reinforcement-based, and implicit use-dependent learning.

## Forging a stronger bond between cognition and action

We have demonstrated the important, yet underappreciated role of explicit strategy use in sensorimotor learning. Consequently, there has been limited progress in the development of models that incorporate this crucial component of motor learning. Here, we present a framework that postulates how successful motor performance relies on three fundamental processes: reasoning, refinement, and retrieval. As these ideas advance toward a formal computational account, we see opportunities for increased cross-pollination between motor learning and cognitive science communities. Undoubtedly, these intellectual bonds will be essential for developing a comprehensive theory of motor learning, capable of explaining the intricate cognitive–motor interactions that facilitate successful motor skill acquisition.

## Open questions

1. How do reasoning, refinement, and retrieval differ across motor learning tasks? For example, how do action–outcome hypotheses and utility functions differ between skills that are part of our natural development (e.g., reaching, walking) and those that may be acquired at a later age (e.g., knitting, ballroom dancing)?
2. Neuropsychological findings suggest that the prefrontal cortex and cerebellum may play a role in reasoning but not in refinement or retrieval (Butcher et al., 2017; McDougle et al., 2022a; Taylor and Ivry, 2014; Tsay et al., 2023b; Wong et al., 2019). Are other brain areas involved in retrieval but not reasoning (Shmuelof et al., 2014)? More generally, how are reasoning, refinement, and retrieval implemented in the brain?
3. What are the behavioral and neural constraints underlying the transition of motor skills from being deliberate to automatic, from being explicit to implicit (Fresco et al., 2023; Servant et al., 2018)?
4. How can the 3R framework inform physical rehabilitation for patients with movement disorders (Roemmich and Bastian, 2018; Tsay and Winstein, 2021)? How do individual features such as age, physical fitness, and different cognitive abilities impact reasoning, refinement, and retrieval (Anderson et al., 2021; Anguera et al., 2010; Guo and Song, 2023; Tsay et al., 2023a)?
5. How are reasoning, refinement, and retrieval impacted by changes in context (Avraham et al., 2022; Dawidowicz et al., 2022; Forano et al., 2021; Heald et al., 2021)?
6. How do reasoning, recall, and refinement contribute to motor learning in amnesia, as observed in cases like H.M., where explicit memory of the task is absent?
